# Estimating the global prevalence of secondary hyperparathyroidism in patients with chronic kidney disease

**DOI:** 10.3389/fendo.2024.1400891

**Published:** 2024-06-21

**Authors:** Yichao Wang, Jiaye Liu, Yiqiao Fang, Shengliang Zhou, Xueting Liu, Zhihui Li

**Affiliations:** ^1^ Division of Thyroid Surgery, Department of General Surgery, West China Hospital, Sichuan University, Chengdu, Sichuan, China; ^2^ Laboratory of Thyroid and Parathyroid Diseases, Frontiers Science Center for Disease-Related Molecular Network, West China Hospital, Sichuan University, Chengdu, Sichuan, China; ^3^ West China School of Medicine, West China Hospital, Sichuan University, Chengdu, Sichuan, China; ^4^ Department of Evidence-Based Medicine and Clinical Epidemiology, West China Hospital, Sichuan University, Chengdu, Sichuan, China

**Keywords:** secondary hyperparathyroidism, prevalence, chronic kidney disease, meta-analysis, meta

## Abstract

**Background:**

Chronic kidney disease (CKD)-related secondary hyperparathyroidism (SHPT) is associated with higher morbidity and death. The goal of this study was to mine the SHPT data already available to do a meta-analysis on the global prevalence of SHPT caused by CKD.

**Methods:**

Embase, Medline, Web of Science, Cochrane Central Databases, and Google Scholar were searched to identify studies on the prevalence of SHPT due to CKD from inception to November 2023. Pooled prevalence was calculated using the DerSimonian-Laird random effects model with a logit transformation.

**Results:**

Twenty-one eligible studies involving 110977 patients were included. Our results revealed that the estimated global prevalence of SHPT due to CKD was 49.5% (95% CI 30.20–68.18), regardless of the diagnostic criteria. For subgroup analysis, Southern Asia (84.36%, 95% CI 79.35–88.34) had a significantly higher SHPT prevalence than other geographic regions. SHPT due to CKD was most prevalent in China (85.14%, 95% CI 81.74–88.00).

**Conclusions:**

SHPT due to CKD is highly prevalent. This necessitates awareness and therapeutic approaches from primary care physicians, medical professionals, and health strategy authorities.

**Systematic Review Registration:**

https://www.crd.york.ac.uk/PROSPERO, identifier CRD42024514007.

## 1 Introduction

Hyperparathyroidism (HPT) is classified as primary, secondary, and tertiary. The causes of secondary hyperparathyroidism (SHPT) include chronic kidney disease (CKD), deficiencies of vitamin D, rickets, and other factors (1, 2). CKD, considered an abnormality of kidney structure or function, is widely recognized as the leading cause of SHPT. It is evident that the prevalence of CKD in the general population is rapidly growing. SHPT, as one of the common comorbidities associated with advanced CKD, has become almost universal in CKD patients (2). SHPT, characterized by elevated parathyroid hormone (PTH), hypocalcemia, and hyperphosphatemia, affects cardiovascular, nervous, skeletal, blood, and other systems. As a result, SHPT may contribute to the development of bone demineralization, high bone turnover, and extraskeletal calcification (3). Patients with SHPT may suffer from severe cardiovascular calcification, renal osteopathy, fractures, erythropoietin resistance, and other adverse clinical events (3). Increasing evidence suggests that SHPT patients are more likely to suffer from worse quality of life, increased cardiovascular morbidity, and mortality (4, 5). Alarmingly, SHPT has become a major public health issue associated with the enormous consumption of economic and social resources (6, 7).

It has been demonstrated that the prevalence of SHPT resulting from CKD varies widely, ranging from 31% to 85% (6, 8, 9). A systematic review has documented that the prevalence of SHPT due to CKD ranges from 30% to 54% in Europe, Australia, and the Americas, but this rate reaches as low as 11.5%–28% in Asia (10). However, to date, there is a paucity of meta-analysis that assesses SHPT prevalence in CKD patients. To address this issue, we conducted a systematic review and meta-analysis to evaluate the global prevalence of SHPT due to CKD by exploring the existing epidemiologic data on SHPT due to CKD.

## 2 Materials and methods

This study was conducted entirely in compliance with the PRISMA declaration, and this protocol has been registered with the PROSPERO International Prospective Register of Systematic Reviews of the University of York (CRD42024514007).

### 2.1 Systematic literature search

Embase, Medline, Web of Science, Cochrane Central Databases, and Google Scholar were searched systematically for all articles published in the English language up until November 2023. The following terms were used: “secondary hyperparathyroidism,” “chronic kidney disease,” “epidemiology,” and “prevalence” (the full search strategies are provided in the **Supplementary Methods**). To locate pertinent research, reference lists of earlier papers were also found. Only human studies with full-text descriptions that were published in English were considered. When two independent reviewers could not agree on whether or not an article should be included, a third reviewer was chosen to make the final decision.

### 2.2 Inclusion and exclusion criteria

Two reviewers independently screened and identified the search findings for eligible studies. Inclusion criteria were as follows: (1) studies identified SHPT; (2) patients with CKD; (2) studies reported clear documentation of the prevalence of SHPT; (3) study period from January 1990 to November 2023. Studies were excluded from the analysis based on the following: (1) case reports, abstracts, reviews, correspondence, letters, editorials, and expert opinions; (2) studies with no clear data for authors to calculate the aggregated prevalence; and (3) studies that were not reported in English.

### 2.3 Data extraction and quality assessment

Data were extracted by three independent reviewers from eligible studies using standardized forms, with 10% of studies randomly checked by another author. When similar studies were published by the same institution or authors, either the largest sample size or the most recent publication was included in the analysis. The recorded data included: year of publication, study year, first author, country or region, level of country development, sex, mean age, diagnostic criteria, prevalence of disease, number of patients, study design, and study source. The quality of the included study was assessed using the Newcastle Ottawa scale (NOS) by examining three factors: patient selection, comparability of the two groups, and assessment of outcome (11). Studies valued at six or more stars were considered to be of higher quality. Based on the quality score, studies were not excluded for improving transparency and ensuring available data were reported.

### 2.4 Statistical analysis

Meta-analysis was performed using the “Meta” and “Metafor” modules in the R-4.0.0 statistical software package. A 95% confidence interval (CI) was calculated using the Wilson score method, and pooled prevalence was analyzed using the DerSimonian-Laird random-effects model with logit transformation. Heterogeneity was measured using the Cochran Q statistics and *I^2^* statistics. Pooled prevalence was calculated using a random-effects model because global evidence was expected to be heterogeneous. Additionally, sensitivity analyses were undertaken by leave-one-out diagnostic tests, and findings were verified by a built-in function. Subgroup analyses were also undertaken to evaluate potential heterogeneity. *P* values were used for the evaluation of the difference between subgroup analyses. *P* values <0.05 indicated a significant difference.

## 3 Results

### 3.1 Study characteristics

**Figure 1:**
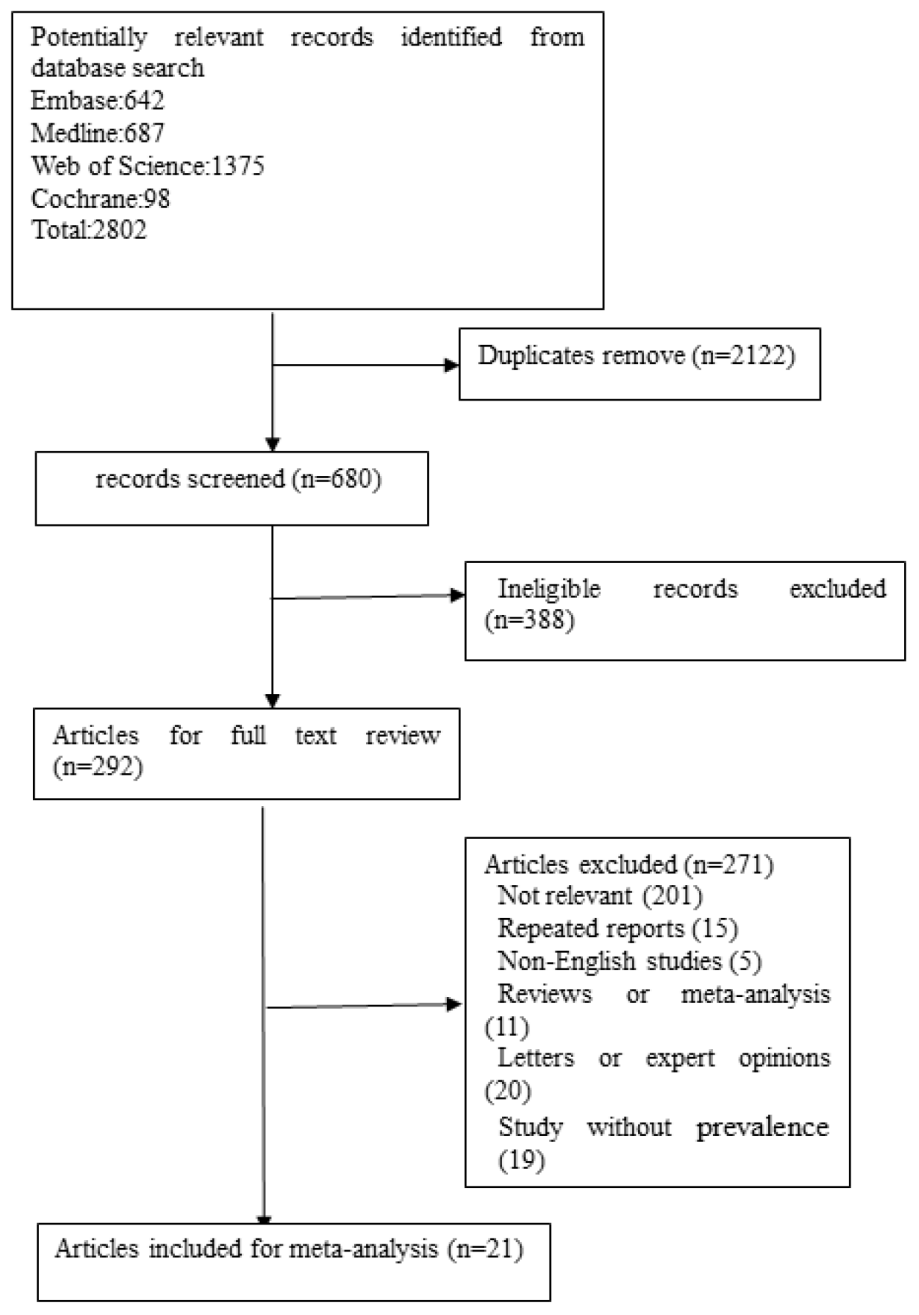
Flow diagram depicting the study selection.

The search generated 680 records after removing duplicates. Initial screening of titles and abstracts resulted in the exclusion of 388 records. The full text of 292 articles was selected for further investigation. Finally, 21 studies (6, 8, 9, 12–29) matched our inclusion criteria and were included in the analysis (**Figure 1**). The general characteristics of the included studies in the meta-analysis are shown in **Table 1**. The quality assessment scores for the included studies are given in **Table 2**. The quality scores of all studies ranged from 6 to 8. As a result, the majority of the included studies had a cross-sectional design. The mean or median age of participants ranged from 44.47 years to 65.40 years. The percentages of women ranged from 18.75% to 53.30%.

**Table 1:** Characteristics of the studies included in the meta-analysis.

Study	Country	Year	Study time	Design	Sample source	Diagnostic criteria	Age	CKD stage	SHPT	Individuals
Xu Y (6)	Sweden	2021	2006–2011	Cross-sectional	School	iPTH > 65 pg/mL	65.4 ±14.5	stage 1–5	784	2,556
Okoye JU (8)	Nigeria	2015	2010–2011	Cross-sectional	Hospital	iPTH > 65 pg/mL	Not specified	Not specified	72	85
Owda A (9)	USA	2016	2013	Cross-sectional	Hemodialysis center	iPTH > 200 pg/mL	58 ± 14	Not specified	95	122
Arévalo-Lorido JC (12)	Spain	2016	2013	Cross-sectional	Community	iPTH ≥ 70 pg/mL	Not specified	stage 3 and 4	275	409
Gimba ZM (13)	Nigeria	2018	2011–2012	Cross-sectional	Hospital	iPTH ≥ 65 pg/mL	44.17 ± 15.60	stage 2–5	127	230
Abdu A (14)	Nigeria	2019	2011–2012	Cross-sectional	Hospital	iPTH > 400 pg/mL	45.96 ± 13.7	Not specified	15	48
Sutton W (15)	USA	2022	2008–2020	Prospective	Hospital	iPTH ≥ 70 pg/mL	53.1 ± 13.7	Not specified	524	849
Seck SM (16)	Senegal	2012	2011	Cross-sectional	Dialysis center	Patients with high turn-over osteopathy	Not specified	Not specified	57	118
Gutiérrez OM (17)	USA	2008	2004	Cross-sectional	SEEK	iPTH > 65 pg/mL	Not specified	Not specified	103	1,860
Căpuşă C (18)	Romania	2016	Not specified	Cross-sectional	Tertiary care center	iPTH > 73 pg/mL	Not specified	stage 2–5	65	115
Salem MM (19)	USA	1997	2012–2013	Cross-sectional	Outpatient dialysis units	iPTH > 195 pg/mL	56 ± 15	Not specified	305	612
Ghosh B (20)	India	2012	2008–2010	Cross-sectional	Hospital	iPTH > 69 pg/mL	45.67 ± 16.96	stage 4 and 5	131	150
Vikrant S1	India	2016	2011–2014	Cross-sectional	Community	iPTH > 65 pg/mL	56.8 ± 13.1	stage 3 and 5	382	462
Rahimian M (22)	Iran	2008	2009–2010	Cross-sectional	Hospital	Not specified	Not specified	Not specified	36	80
Bhan I (23)	USA	2010	2002–2007	Retrospective	MGPC network	iPTH > 60 pg/mL	Not specified	stage 3 and 4	688	1,252
Chua CC (24)	Philippine	2010	2000–2009	Cross-sectional	Community	Not specified	64 ± 11	Not specified	41	142
Schumock GT (25)	USA	2008	2000–2004	Retrospective	PharMetrics Patient-centric database	based on at least one medical or facility claim indicative of the diagnosis, ICD-9 code 588.8x	Not specified	Not specified	667	66,019
Jovanovich A (26)	USA	2012	2001–2003	prospective	Medical centers	iPTH > 65 pg/mL	Not specified	Not specified	1,285	1,497
Lou I (27)	Nigeria	2015	2004–2012	Cross-sectional	Hospital	iPTH > 72 pg/mL	51.5 ± 0.51	Not specified	694	1,609
Oliveira RB (28)	Brazil	2011	2010–2011	Cross-sectional	Dialysis units	iPTH > 1,000 pg/mL	Not specified	Not specified	3,463	32,264
Wei Y (29)	China	2016	2008–2012	Cross-sectional	School	iPTH > 88 pg/mL	49.45 ± 16.33	Not specified	424	498

iPTH, intact parathyroid hormone; SHPT, secondary hyperparathyroidism; CKD, chronic kidney disease; MGPC, Massachusetts general hospital primary care; and ICD, international classification of diseases.

**Table 2:** Newcastle-Ottawa scoring for including studies.

	Year	Representative (0–1)	Present outcome at the beginning of study (0,1)	Sample size (0, 1)	Diagnostic tool (0–2)	Comparability of study population (0 or 2)	Outcome assessment (0–1)	Statistical test (0–1)	Score
Xu Y (6)	2021	1	1	1	1	2	1	1	8
Okoye JU (8)	2015	1	0	1	1	2	1	1	7
Owda A (9)	2016	1	1	1	1	2	1	1	8
Arévalo-Lorido JC (12)	2016	1	1	1	1	2	1	1	8
Gimba ZM (13)	2018	1	0	1	1	1	1	1	6
Abdu A (14)	2019	1	1	1	1	2	1	1	8
Sutton W (15)	2022	1	1	1	1	2	1	1	8
Seck SM (16)	20127	1	0	1	1	1	1	1	6
Gutiérrez OM (17)	2008	1	1	1	1	2	0	1	7
Căpuşă C (18)	2016	1	1	1	1	2	1	1	8
Salem MM (19)	1997	1	1	1	1	1	1	1	7
Ghosh B (20)	2012	1	1	1	1	1	1	1	7
Vikrant S (21)	2016	1	0	1	1	1	1	1	6
Rahimian M (22)	2008	1	1	1	1	2	1	1	8
Bhan I (23)	2010	1	0	1	1	2	1	1	7
Chua CC (24)	2010	1	0	1	0	2	1	1	6
Schumock GT (25)	2008	1	0	1	1	2	0	1	6
Jovanovich A (26)	2012	1	1	1	1	2	0	1	7
Lou I (27)	2015	1	1	1	1	2	1	1	8
Oliveira RB (28)	2011	1	1	1	1	1	1	1	7
Wei Y (29)	2016	1	0	1	1	2	1	1	7

### 3.2 SHPT prevalence

**Figure 2:**
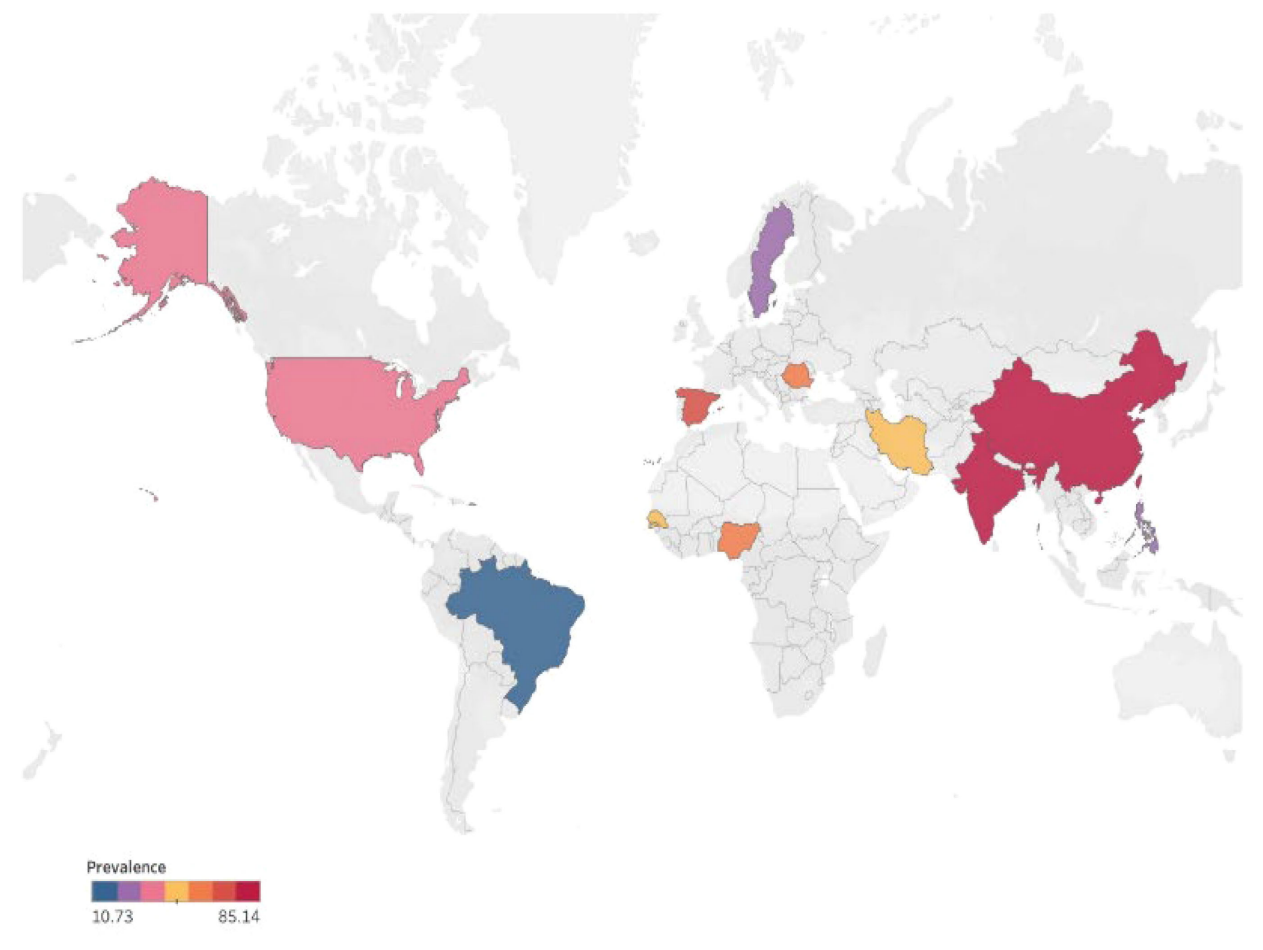
Global prevalence of secondary hyperparathyroidism in patients with chronic kidney disease.

**Figure 3:**
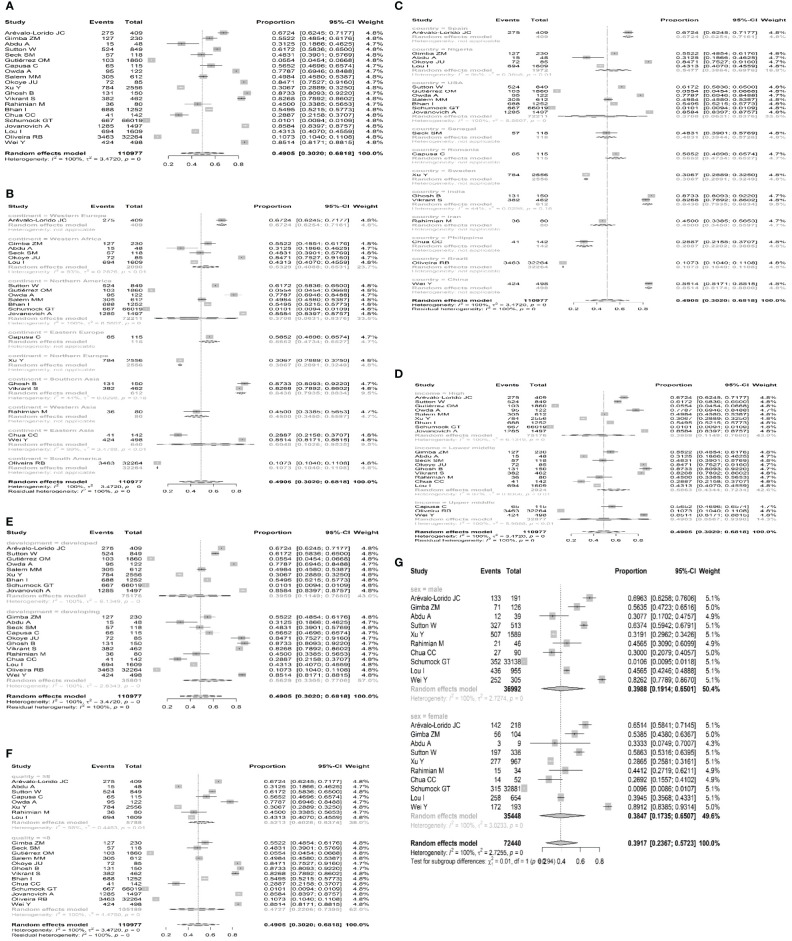
**(A)** Forest plots displaying prevalence in CKD patients. SHPT, secondary hyperparathyroidism; CKD, chronic kidney disease; and CI, confidence intervals. **(B)** Forest plots displaying the prevalence of SHPT stratified by continents. SHPT, secondary hyperparathyroidism; CKD, chronic kidney disease; and CI, confidence intervals. **(C)** Forest plots displaying the prevalence of SHPT stratified by countries and regions. SHPT, secondary hyperparathyroidism; CKD, chronic kidney disease; and CI, confidence intervals. **(D)** Forest plots displaying the prevalence of SHPT stratified by country income. SHPT, secondary hyperparathyroidism; CKD, chronic kidney disease; and CI, confidence intervals. **(E)** Forest plots displaying the prevalence of SHPT stratified by the development of countries. SHPT, secondary hyperparathyroidism; CKD, chronic kidney disease; and CI, confidence intervals. **(F)** Forest plots displaying the prevalence of SHPT stratified by study quality. SHPT, secondary hyperparathyroidism; CKD, chronic kidney disease; and CI, confidence intervals. **(G)** Forest plots displaying the prevalence of SHPT stratified by sex. SHPT, secondary hyperparathyroidism; CKD, chronic kidney disease; and CI, confidence intervals.

Overall, 21 studies from five countries or regions (three European countries, five African countries, seven Northern American countries, five Asian countries and regions, and one South American country) involved a total of 110,977 individuals who reported SHPT prevalence in patients with chronic kidney disease. A total of 10,233 participants were diagnosed with SHPT. The overall pooled estimated prevalence was 49.5% (95% CI 30.20 – 68.18, I2 = 100%, **Figures 2**, **3A**), regardless of the diagnostic criteria. By performing sensitivity analysis and leave-one-out analysis, we failed to identify outliers. (**Supplementary Tables 1**, **2**; **Supplementary Figure 1**). sAfter conducting subgroup analysis, varied prevalences of SHPT were observed across different geographic regions (*P* < 0.05). The highest SHPT prevalence was found in Southern Asia with an estimated rate of 84.36% (95% CI 79.35–88.34, **Figure 3B**), followed by Western Europe (67.24%, 95% CI 62.54–71.61), Eastern Asia (60.48%, 95% CI 10.26–95.35), Eastern Europe (56.52%, 95% CI 47.34–65.27), Western Africa (53.29%, 95% CI 40.88–65.31), Western Asia (45.00%, 95% CI 34.50–55.97), Northern America (37.08%, 95% CI 6.31–83.76), Northern Europe (30.67%, 95% CI 28.911–32.49), and South America (10.73%, 95% CI 10.40–11.08). By stratifying data according to countries and regions, SHPT prevalence varied from 10.73% (Brazil, 95% CI 10.40–11.08, **Figure 3C**) to 85.14% (Chin, 95% CI 81.74–88.00, **Figure 3C**) (*P* < 0.05). Considering the country income, SHPT prevalence was higher in lower-middle-income countries (58.63%, 95% CI 43.44–72.34, **Figure 3D**) than in high-income countries (39.59%, 95% CI 11.49–76.80, **Figure 3D**) or upper-middle-income countries (49.03%, 95% CI 5.67–93.90, **Figure 3D**), although without a significant difference. The SHPT prevalence was 56.29% (95% CI, 33.05%–53.3%, **Figure 3E**) and 39.59% (95% CI, 11.49%–76.80%, **Figure 3E**) in developing and developed countries, respectively. Moreover, SHPT prevalence for the quality scores of studies valued above or below 8 points was 52.13% (95% CI 40.28–63.74, **Figure 3F**) and 47.27% (95% CI 22.06–73.95, **Figure 3F**), respectively. When stratifying data by sex, the SHPT prevalence was 39.88% (95% CI 19.14–65.01, **Figure 3G**) in men and 38.47% (95% CI 17.35–65.07, **Figure 3G**) in women.

## 4 Discussion

To the best of our knowledge, this is the first comprehensive meta-analysis aimed at estimating the global prevalence of SHPT due to CKD. In our meta-analysis, we estimated the global prevalence of SHPT due to CKD through existing epidemiology data. We found that the overall prevalence of SHPT due to CKD is 49.5% (95% CI 30.20–68.18), regardless of the diagnostic criteria. More importantly, our results revealed significant differences among geographic regions and countries, with Southern Asia and China having the highest prevalence rates (84.36%, 95% CI 79.35–88.34) and (85.14%, 95% CI 81.74–88.00), respectively.

There has been one systematic review showing that SHPT prevalence among dialysis populations is highly variable based on geographic regions. In Europe and Australia, SHPT prevalence varied from 30% to 49% (10). SHPT prevalence in the Americas was estimated at 54%, while that in Asia (India and Japan) was 28% and 11.5%, respectively (10). In contrast, we observed that Southern Asia led with the highest prevalence (84.36%), followed by Western Europe (67.24%). SHPT prevalence was lowest in South America (10.73%). This discrepancy might be attributed to differences in cutoff values for diagnostic criteria, cultural traditions, population selection, and duration of dialysis (29). In addition, underdiagnosis in lower-middle-income geographic regions may be a factor contributing to the discrepancy.

It is evident that sex is considered one of the risk factors for SHPT, though the effect of sex remains debated. A previous study documented that the female sex is associated with the development of SHPT in CKD patients (13). However, Xu et al. reported that the male sex is correlated with a higher risk of SHPT due to CKD (6). Unexpectedly, in the current study, we observed a comparable prevalence of SHPT between men and women. Further prospective trials with larger sample sizes are needed to confirm the effects of sex on SHPT.

It has been demonstrated that various factors are correlated with the development of SHPT. Hyperphosphatemia, hypocalcemia, and elevated alkaline phosphatase levels are identified as risk factors for SHPT (13). Additionally, a low estimated glomerular filtration rate, young age, male sex, and diabetes are strong risk factors for SHPT development (6). Previous studies have also highlighted that serum creatinine and phosphorus are independently associated with SHPT (29). Unfortunately, we cannot calculate pooled estimates of the risk factors associated with SHPT prevalence due to the limited number of studies.

Nevertheless, the current study has several limitations. First, a relatively small number of studies were included in the meta-analysis. Second, limited data from Western Europe, Eastern Europe, Northern Europe, Western Asia, and South America made it challenging to accurately estimate SHPT prevalence in these continents. Third, the different cutoff values for diagnostic criteria could result in publication bias. Additionally, this study failed to pool and estimate some stratified data due to the limited number of studies on CKD stage, dialysis vintage, co-morbidities such as diabetics, hypertension, and race.

## 5 Conclusion

In summary, this systematic review demonstrates a high prevalence of SHPT in patients with CKD. The significant threat to public health and growing burden on SHPT patients are more worrisome. Our findings call for increased attention and management for SHPT in CKD patients from primary care physicians, medical professionals, and health strategy authorities. More emphasis should also be placed on improving the diagnosis of SHPT. However, there is a need for further adequately powered, well-designed prospective studies to clarify the global epidemiology of SHPT in CKD patients.

## Data availability statement

The datasets presented in this study can be found in online repositories. The names of the repository/repositories and accession number(s) can be found in the article/**Supplementary Material**.

## Author contributions

ZL: Resources, Project administration, Writing – review & editing, Visualization, Validation, Supervision, Methodology, Conceptualization. YW: Writing – original draft, Visualization, Software, Resources, Project administration, Methodology, Investigation, Formal analysis, Conceptualization. JL: Writing – original draft, Resources, Project administration, Methodology, Investigation, Formal analysis, Data curation. YF: Writing – original draft, Validation, Software, Resources, Methodology, Investigation, Funding acquisition, Formal analysis, Data curation. SZ: Writing – review & editing, Validation, Methodology, Formal analysis, Conceptualization. XL: Writing – review & editing, Visualization, Validation, Supervision, Methodology, Data curation.
