# Supplementary material for: Estimating the global prevalence of secondary hyperparathyroidism in patients with chronic kidney disease
Source: Front Endocrinol (Lausanne). 2024 Jun 21;15:1400891. doi: 10.3389/fendo.2024.1400891 (PMC11224516; doi:10.3389/fendo.2024.1400891)
Supplement: Supplementary file 1 [file DataSheet_1.docx]

**Supplementary Methods: Searching strategy for prevalence** **of secondary hyperparathyroidism in patients with chronic kidney disease**

| **Database searched** | **Records** | **Records after duplicates removed** |
| --- | --- | --- |
| embase.com | 642 | 601 |
| Medline Ovid | 687 | 588 |
| Web of science Core Collection | 1375 | 876 |
| Cochrane CENTRAL | 98 | 57 |
| **Overall** | **2802** | **2122** |

**Embase.** **com**

(('epidemiological data'/de OR 'epidemiology'/de OR 'geographic distribution'/de OR 'patient volume'/de OR prevalence/exp OR 'incidence'/de OR geography/de OR 'geographic names'/exp OR 'cross-sectional study'/de OR (epidemiolog* OR ((geograph* OR global*) NEAR/3 (distribut*)) OR (patient* NEAR/3 volume*) OR prevalen* OR incidenc* OR population-based* OR cross-sectional*):ab,ti,kw) NOT ((animal/exp OR animal*:de OR nonhuman/de) NOT ('human'/exp)) NOT ('case report'/de OR 'case report*':ti) NOT ([Conference Abstract]/lim) AND [english]/lim) AND ('secondary hyperparathyroidism'/de OR ('hyperparathyroidism, secondary' OR 'secondary parathyroidism' OR 'secondary hyperparathyroidism'):ab,ti,kw) AND ('chronic kidney failure'/de OR ('chronic kidney disease' OR 'chronic kidney disorder' OR 'chronic kidney insufficiency' OR 'chronic nephropathy' OR 'chronic renal disease' OR 'chronic renal failure' OR 'chronic renal insufficiency' OR 'kidney chronic failure' OR 'kidney disease, chronic' OR 'kidney failure, chronic' OR 'kidney function, chronic disease' OR 'renal insufficiency, chronic' OR 'chronic kidney failure'):ab,ti,kw)

**Medline** **Ovid**

((Epidemiological Monitoring/ OR Epidemiology/ OR Epidemiology.fs. OR exp Incidence/ OR exp Prevalence/ OR Incidence/ OR Geography/ OR exp Geographic Locations/ OR Epidemiologic Studies OR Cross-Sectional Studies/ OR (epidemiolog* OR ((geograph* OR global*) ADJ3 (distribut*)) OR (patient* ADJ3 volume*) OR prevalen* OR incidenc* OR population-based* OR cross-sectional*).ab,ti,kf.) NOT (exp Animals/ NOT Humans/) NOT (news OR congres* OR abstract* OR book* OR chapter* OR dissertation abstract*).pt. AND english.la.) AND AB,TI,KF('secondary hyperparathyroidism'/ OR ('hyperparathyroidism, secondary' OR 'secondary parathyroidism' OR 'secondary hyperparathyroidism')) AND AB,TI,KF('chronic kidney failure'/ OR ('chronic kidney disease' OR 'chronic kidney disorder' OR 'chronic kidney insufficiency' OR 'chronic nephropathy' OR 'chronic renal disease' OR 'chronic renal failure' OR 'chronic renal insufficiency' OR 'kidney chronic failure' OR 'kidney disease, chronic' OR 'kidney failure, chronic' OR 'kidney function, chronic disease' OR 'renal insufficiency, chronic' OR 'chronic kidney failure'))

**Web of science Core Collection**

TS=((epidemiolog* OR ((geograph* OR global*) NEAR/2 (distribut*)) OR (patient* NEAR/2 volume*) OR prevalen* OR incidenc* OR population-based* OR cross-sectional*) NOT ((animal* OR rat OR rats OR mouse OR mice OR murine OR dog OR dogs OR canine OR cat OR cats OR feline OR rabbit OR cow OR cows OR bovine OR rodent* OR sheep OR ovine OR pig OR swine OR porcine OR veterinar* OR chick* OR zebrafish* OR baboon* OR nonhuman* OR primate* OR cattle* OR goose OR geese OR duck OR macaque* OR avian* OR bird* OR fish*) NOT (human* OR patient* OR women OR woman OR men OR man))) AND TS=('secondary hyperparathyroidism' OR 'hyperparathyroidism, secondary' OR 'secondary parathyroidism') AND TS=('chronic kidney failure' OR 'chronic kidney disease' OR 'chronic kidney disorder' OR 'chronic kidney insufficiency' OR 'chronic nephropathy' OR 'chronic renal disease' OR 'chronic renal failure' OR 'chronic renal insufficiency' OR 'kidney chronic failure' OR 'kidney disease, chronic' OR 'kidney failure, chronic' OR 'kidney function, chronic disease' OR 'renal insufficiency, chronic')

**Cochrane CENTRAL**

((epidemiolog* OR ((geograph* OR global*) NEAR/3 (distribut*)) OR (patient* NEAR/3 volume*) OR prevalen* OR incidenc* OR (population NEXT/1 based*) OR (cross NEXT/1 sectional*)):ab,ti,kw) AND (('secondary hyperparathyroidism' OR 'hyperparathyroidism, secondary' OR 'secondary parathyroidism'):ab,ti,kw) AND (('chronic kidney failure' OR 'chronic kidney disease' OR 'chronic kidney disorder' OR 'chronic kidney insufficiency' OR 'chronic nephropathy' OR 'chronic renal disease' OR 'chronic renal failure' OR 'chronic renal insufficiency' OR 'kidney chronic failure' OR 'kidney disease, chronic' OR 'kidney failure, chronic' OR 'kidney function, chronic disease' OR 'renal insufficiency, chronic'):ab,ti,kw)

**Supplementary Table 1. Sensitivity analysis and leave-one-out results performed in Metafor package.**

|  | **resid** | **se** | **z** |
| --- | --- | --- | --- |
| 17 | −0.7348 | 0.3679 | −1.9972 |
| 6 | −0.5904 | 0.3282 | −1.7988 |
| 18 | 0.4042 | 0.2887 | 1.4001 |
| 12 | 0.4232 | 0.3204 | 1.3209 |
| 21 | 0.3929 | 0.3119 | 1.2595 |
| 10 | 0.3813 | 0.3239 | 1.1771 |
| 13 | 0.3578 | 0.3135 | 1.1413 |
| 20 | −0.4901 | 0.5100 | −0.9610 |
| 8 | 0.2920 | 0.3223 | 0.9060 |
| 16 | −0.2427 | 0.3238 | −0.7494 |
| 11 | −0.2238 | 0.3225 | −0.6941 |
| 3 | −0.2125 | 0.3289 | −0.6462 |
| 1 | 0.1690 | 0.3178 | 0.5317 |
| 4 | 0.1087 | 0.3457 | −2.3828 |
| 7 | 0.1079 | 0.3234 | 0.3336 |
| 19 | −0.0877 | 0.3174 | −0.2764 |
| 14 | −0.0673 | 0.3255 | −0.2067 |
| 2 | 0.0392 | 0.3213 | 0.1221 |
| 15 | 0.0367 | 0.3140 | 0.1168 |
| 5 | −0.0330 | 0.3238 | 0.1020 |
| 9 | −0.0171 | 0.3191 | −0.0536 |

|  | **estimate** | **zval** | **pval** | **ci.lb** | **ci.ub** | **Q** | **Qp** | **tau2** | ***I*^2^** | **H2** |
| --- | --- | --- | --- | --- | --- | --- | --- | --- | --- | --- |
| 1 | 0.5066 | 11.3863 | 0 | 0.3711 | 0.6416 | 23555.6569 | 0 | 0.0955 | 99.9193 | 1239.7714 |
| 2 | 0.5129 | 11.3776 | 0 | 0.3760 | 0.6488 | 24075.5503 | 0 | 0.0973 | 99.9211 | 1267.1342 |
| 3 | 0.5245 | 11.5075 | 0 | 0.3869 | 0.6603 | 24376.8827 | 0 | 0.0981 | 99.9221 | 1282.9938 |
| 4 | 0.5095 | 11.5255 | 0 | 0.3750 | 0.6433 | 22910.7309 | 0 | 0.0939 | 99.9171 | 1205.8279 |
| 5 | 0.5163 | 11.3986 | 0 | 0.3790 | 0.6524 | 24270.4580 | 0 | 0.0978 | 99.9217 | 1277.3925 |
| 6 | 0.5433 | 11.5082 | 0 | 0.4022 | 0.6809 | 24400.8802 | 0 | 0.1024 | 99.9221 | 1284.2569 |
| 7 | 0.5096 | 11.3208 | 0 | 0.3727 | 0.6458 | 24200.2568 | 0 | 0.0975 | 99.9215 | 1273.6977 |
| 8 | 0.5009 | 11.2280 | 0 | 0.3647 | 0.6370 | 24059.5337 | 0 | 0.0969 | 99.9210 | 1266.2912 |
| 9 | 0.5156 | 11.4565 | 0 | 0.3791 | 0.6509 | 23682.8768 | 0 | 0.0965 | 99.9198 | 1246.4672 |
| 10 | 0.4968 | 11.1654 | 0 | 0.3607 | 0.6332 | 24111.6504 | 0 | 0.0971 | 99.9212 | 1269.0342 |
| 11 | 0.5256 | 11.4604 | 0 | 0.3872 | 0.6620 | 23168.0730 | 0 | 0.0989 | 99.9180 | 1219.3723 |
| 12 | 0.4946 | 11.1829 | 0 | 0.3591 | 0.6304 | 23846.2378 | 0 | 0.0961 | 99.9203 | 1255.0651 |
| 13 | 0.4975 | 11.4051 | 0 | 0.3640 | 0.6311 | 22906.8079 | 0 | 0.0930 | 99.9171 | 1205.6215 |
| 14 | 0.5179 | 11.4167 | 0 | 0.3805 | 0.6539 | 24323.0924 | 0 | 0.0979 | 99.9219 | 1280.1628 |
| 15 | 0.5129 | 11.5916 | 0 | 0.3785 | 0.6464 | 22630.3945 | 0 | 0.0936 | 99.9160 | 1191.0734 |
| 16 | 0.5263 | 11.5191 | 0 | 0.3885 | 0.6621 | 24340.8154 | 0 | 0.0981 | 99.9219 | 1281.0955 |
| 17 | 0.5503 | 10.3596 | 0 | 0.3924 | 0.7032 | 11055.1794 | 0 | 0.1289 | 99.8281 | 581.8515 |
| 18 | 0.4950 | 12.3098 | 0 | 0.3716 | 0.6187 | 19026.6515 | 0 | 0.0792 | 99.9001 | 1001.4027 |
| 19 | 0.5190 | 11.5518 | 0 | 0.3829 | 0.6536 | 22941.2295 | 0 | 0.0957 | 99.9172 | 1207.4331 |
| 20 | 0.5387 | 7.3853 | 0 | 0.3229 | 0.7472 | 22927.8313 | 0 | 0.2477 | 99.9171 | 1206.7280 |
| 21 | 0.4958 | 11.4346 | 0 | 0.3630 | 0.6289 | 22667.7481 | 0 | 0.0921 | 99.9162 | 1193.0394 |

**Supplementary Table 2**. **Leave-one-out diagnostics with a built-in function in SHPT prevalence in CKD patients.**

|  | **rstudent** | **dffits** | **cook.d** | **cov.r** | **tau2.del** | **QE.del** | **hat** | **weight** | **dfbs** |
| --- | --- | --- | --- | --- | --- | --- | --- | --- | --- |
| 1 | 0.5317 | 0.1195 | 0.0139 | 1.0239 | 0.0955 | 23555.6569 | 0.0479 | 4.7894 | 0.1195 |
| 2 | 0.1221 | 0.0274 | 0.0007 | 1.0417 | 0.0973 | 24075.5503 | 0.0477 | 4.7664 | 0.0274 |
| 3 | -0.6462 - | 0.1416 | 0.0200 | 1.0482 | 0.0981 | 24376.8827 | 0.0458 | 4.5784 | -0.1416 |
| 4 | 0.3457 | 0.0781 | 0.0058 | 1.0066 | 0.0939 | 22910.7309 | 0.0480 | 4.8048 | 0.0781 |
| 5 | -0.1020 | -0.0227 | 0.0005 | 1.0468 | 0.0978 | 24270.4580 | 0.0472 | 4.7177 | -0.0227 |
| 6 | -1.7988 | -0.4047 | 0.1711 | 1.0970 | 0.1024 | 24400.8802 | 0.0481 | 4.8126 | -0.4048 |
| 7 | 0.3336 | 0.0742 | 0.0055 | 1.0437 | 0.0975 | 24200.2568 | 0.0472 | 4.7151 | 0.0743 |
| 8 | 0.9060 | 0.2018 | 0.0403 | 1.0380 | 0.0969 | 24059.5337 | 0.0472 | 4.7209 | 0.2018 |
| 9 | -0.0536 | -0.0119 | 0.0001 | 1.0343 | 0.0965 | 23682.8768 | 0.0480 | 4.7992 | -0.0119 |
| 10 | 1.1771 | 0.2609 | 0.0674 | 1.0388 | 0.0971 | 24111.6504 | 0.0468 | 4.6796 | 0.2609 |
| 11 | -0.6941 | -0.1562 | 0.0246 | 1.0595 | 0.0989 | 23168.0730 | 0.0481 | 4.8144 | -0.1562 |
| 12 | 1.3209 | 0.2948 | 0.0852 | 1.0297 | 0.0961 | 23846.2378 | 0.0474 | 4.7389 | 0.2948 |
| 13 | 1.1413 | 0.2567 | 0.0625 | 0.9973 | 0.0930 | 22906.8079 | 0.0479 | 4.7927 | 0.2566 |
| 14 | -0.2067 | -0.0457 | 0.0021 | 1.0477 | 0.0979 | 24323.0924 | 0.0467 | 4.6712 | -0.0457 |
| 15 | 0.1168 | 0.0267 | 0.0007 | 1.0038 | 0.0936 | 22630.3945 | 0.0481 | 4.8094 | 0.0267 |
| 16 | -0.7494 | -0.1671 | 0.0279 | 1.0506 | 0.0981 | 24340.8154 | 0.0473 | 4.7345 | -0.1671 |
| 17 | -1.9972 | -0.4500 | 0.2662 | 1.3768 | 0.1289 | 11055.1794 | 0.0482 | 4.8190 | -0.4507 |
| 18 | 1.4001 | 0.3180 | 0.0817 | 0.8507 | 0.0792 | 19026.6515 | 0.0481 | 4.8110 | 0.3176 |
| 19 | -0.2764 | -0.0619 | 0.0037 | 1.0260 | 0.0957 | 22941.2295 | 0.0481 | 4.8116 | -0.0619 |
| 20 | -0.9610 | -0.2182 | 0.1202 | 2.6342 | 0.2477 | 22927.8313 | 0.0482 | 4.8188 | -0.2189 |
| 21 | 1.2595 | 0.2834 | 0.0755 | 0.9879 | 0.0921 | 22667.7481 | 0.0479 | 4.7946 | 0.2833 |

Abbreviations: CKD, chronic kidney disease; SHPT, secondary hyperparathyroidism.

**Supplementary** **Figure 1：Leave-one-out diagnostics with a built-in function in SHPT prevalence in CKD patients**


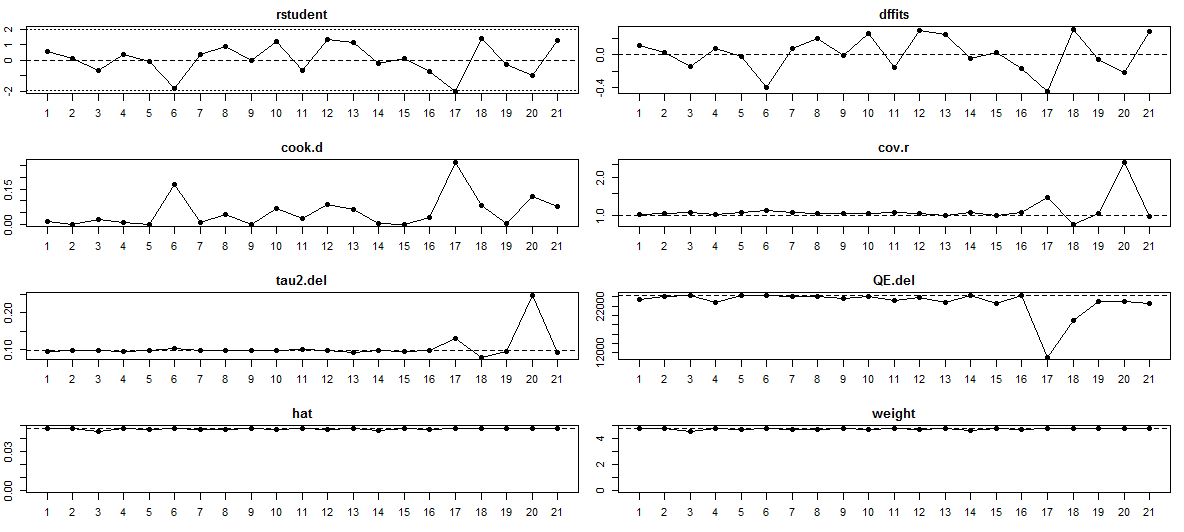


1. Arévalo-Lorido JC, Carretero-Gómez J, García-Sánchez F, Maciá-Botejara E, Ramiro-Lozano JM, Masero-Carretero A, Robles NR, Bureo-Dacal JC. Secondary hyperparathyroidism prevalence and profile, between diabetic and non-diabetic patients with stage 3 to 4 chronic kidney disease attended in internal medicine wards. MiPTH study. Diabetes Metab Syndr. 2016 Apr-Jun;10(2 Suppl 1):S16-21.

2. Gimba ZM, Abene EE, Agbaji OOO, Agaba EI. Secondary hyperparathyroidism among Nigerians with chronic kidney disease. Afr Health Sci. 2018 Jun;18(2):446-457.

3. Abdu A, Abdu A, Arogundade FA. Prevalence and pattern of chronic kidney disease-mineral bone disorders among hemodialysis patients in kano, northwest nigeria. Ann Afr Med. 2019 Oct-Dec;18(4):191-195. doi: 10.4103/aam.aam_18_19.

4. Sutton W, Chen X, Patel P, Karzai S, Prescott JD, Segev DL, McAdams-DeMarco M, Mathur A. Prevalence and risk factors for tertiary hyperparathyroidism in kidney transplant recipients. Surgery. 2022 Jan;171(1):69-76.

5. Seck SM, Dahaba M, Ka EF, Cisse MM, Gueye S, Tal AO. Mineral and bone disease in black african hemodialysis patients: a report from senegal. Nephrourol Mon. 2012 Fall;4(4):613-6. doi: 10.5812/numonthly.4225. Epub 2012 Sep 24.

6. Gutiérrez OM, Isakova T, Andress DL, Levin A, Wolf M. Prevalence and severity of disordered mineral metabolism in Blacks with chronic kidney disease. Kidney Int. 2008 Apr;73(8):956-62.

7. Căpuşă C, Chirculescu B, Vladu I, Viaşu L, Lipan M, Moţa E, Mircescu G. THE PREVALENCE OF BIOCHEMICAL ABNORMALITIES OF CHRONIC KIDNEY DISEASE. MINERAL AND BONE DISORDERS IN UNTREATED NON-DIALYSIS PATIENTS - A MULTICENTER STUDY. Acta Endocrinol (Buchar). 2016 Jul-Sep;12(3):282-290.

8. Owda A, Elhwairis H, Narra S, Towery H, Osama S.Secondary hyperparathyroidism in chronic hemodialysis patients: prevalence and race. Ren Fail. 2003 Jul;25(4):595-602.

9. Salem MM. Hyperparathyroidism in the hemodialysis population: a survey of 612 patients. Am J Kidney Dis. 1997 Jun;29(6):862-5.

10. Okoye JU, Arodiwe EB, Ulasi II, Ijoma CK, Onodugo OD. Prevalence of CKD-MBD in pre-dialysis patients using biochemical markers in Enugu, South-East Nigeria. Afr Health Sci. 2015 Sep;15(3):941-8.

11. Xu Y, Evans M, Soro M, Barany P, Carrero JJ. Secondary hyperparathyroidism and adverse health outcomes in adults with chronic kidney disease. Clin Kidney J. 2021 Jan 20;14(10):2213-2220.

12. Ghosh B, Brojen T, Banerjee S, Singh N, Singh S, Sharma OP, Prakash J. The high prevalence of chronic kidney disease-mineral bone disorders: A hospital-based cross-sectional study. Indian J Nephrol. 2012 Jul;22(4):285-91.

13. Vikrant S, Parashar A. Prevalence and severity of disordered mineral metabolism in patients with chronic kidney disease: A study from a tertiary care hospital in India. Indian J Endocrinol Metab. 2016 Jul-Aug;20(4):460-7.

14. Rahimian M, Sami R, Behzad F. Evaluation of secondary hyperparathyroidism in patients undergoing hemodialysis. Saudi J Kidney Dis Transpl. 2008 Jan;19(1):116-9.

15. Bhan I, Dubey A, Wolf M. Diagnosis and management of mineral metabolism in CKD. J Gen Intern Med. 2010 Jul;25(7):710-6.

16. Chua CC , Rivero W, Gutierez MJ, Jasul GV. Prevalence of Secondary Hyperparathyroidism Among Outpatient Type 2 Diabetic Patients Undergoing Hemodialysis in a Tertiary Hospital. Philippine Journal of Internal Medicine.2010;48(1)

17. Schumock GT, Andress D, E Marx S, Sterz R, Joyce AT, Kalantar-Zadeh K. Impact of secondary hyperparathyroidism on disease progression, healthcare resource utilization and costs in pre-dialysis CKD patients. Curr Med Res Opin. 2008 Nov;24(11):3037-48.

18. Jovanovich A, Chonchol M, Cheung AK, Kaufman JS, Greene T, Roberts WL, Smits G, Kendrick J; HOST Investigators. Racial differences in markers of mineral metabolism in advanced chronic kidney disease.

Clin J Am Soc Nephrol. 2012 Apr;7(4):640-7.

19. Lou I, Foley D, Odorico SK, Leverson G, Schneider DF, Sippel R, Chen H. How Well Does Renal Transplantation Cure Hyperparathyroidism? Ann Surg. 2015 Oct;262(4):653-9.

20. Oliveira RB, Silva EN, Charpinel DM, Gueiros JE, Neves CL, Sampaio Ede A, Barreto Fde C, Karohl C, Ribeiro MC, Moysés RM, Jorgetti V, Carvalho AB. Secondary hyperparathyroidism status in Brazil: Brazilian census of parathyroidectomy. J Bras Nefrol. 2011 Dec;33(4):457-62.

21. Wei Y, Lin J, Yang F, Li X, Hou Y, Lu R, Shi X, Liu Z, Du Y. Risk factors associated with secondary hyperparathyroidism in patients with chronic kidney disease.Exp Ther Med. 2016 Aug;12(2):1206-1212.
